# The effect of captopril on the performance of the control strategies of BJUT-II VAD

**DOI:** 10.1186/s12938-016-0247-1

**Published:** 2016-12-28

**Authors:** Kaiyun Gu, Bin Gao, Yu Chang, Yi Zeng

**Affiliations:** School of Life Science and BioEngineering, Beijing University of Technology, Beijing, 100124 People’s Republic of China

## Abstract

**Background:**

With the development of left ventricular assist device (LVAD), the long-term support has been paid more attention by various researchers. According to previous researches, the combination of LVAD and pharmacological therapy can significantly improve the heart rate recovery and survival rate of patient. However, the effect of pharmacological therapy on the cardiovascular hemodynamic states with LVAD support is still unclear.

**Methods:**

In this study, pharmacokinetic model of captopril is established to describe the relationship between plasma–drug concentration and time. Then, combination model, consisting of pharmacokinetic model of captopril and lumped parameter model of cardiovascular system with BJUT-II VAD support, is established to mimic the effect of pharmacological therapy on cardiovascular hemodynamics. BAI control strategy and HR control strategy for BJUT-II VAD are chosen to evaluate their performance by the combination model.

**Results:**

The simulation results demonstrate that the concentration of captopril could affect the pressure and heart rate by changing the peripheral resistance, and then affect the performance of BJUT-II VAD in a short duration. Under the regulation of control strategies of BJUT-II VAD, the hemodynamic states of cardiovascular system returned to the standard value in 10 s.

**Conclusion:**

This study could provide useful information about how to design coupled strategy of LVAD support and pharmacological therapy.

## Background

Left ventricular assist devices (LVAD) have been widely used in the clinical. However, control strategies are still a significant challenge for researchers. The rotational speed of the rVAD needs to be regulated carefully in response to the change in blood demands of patients. Therefore, several control strategies have been designed. Giridharan et al. [1] proposed a physiological controller using the mean differential pressure between the left ventricle and the aorta to regulate pump speed. Boston et al. [2] designed a hierarchical controller to remain the pulsatility of the pump flow signal by the pump speed. Arndt et al. [3] proposed full or partial assistance mode of LVAD by the gradient of differential pressure pulsatility to classify two specific operating modes. Wu et al. [4] proposed a physiological adaptive controller by calculating the change of peripheral resistance according to intrinsic pump parameters (pump speed and current waveform) to regulate the pump speed. Chang et al. [5] reported a global sliding mode controller for an intra-aortic pump to improve the robustness of the controller. Moscato et al. [6] indicated a control strategy using the afterload of the left ventricle derived from left ventricular pressure and blood flow to maintain the afterload of the left ventricle at a level. Besides the control strategies that directly utilize the left ventricular pressure, flow rate and derived variables as the control variables, there exits another kind of control approach. For instance, Vollkron et al. [7] based on linear mapping of the relationship between the heart rate and rotational speed to regulated the pump speed. Similarly, Song et al. [8] studied the difference in varied support modes of LVAD on cardiovascular system. Gao et al. reported a fuzzy logical controller and a model free adaptive controller (MFAC) to improve the control accuracy [9–11]. Both control strategies used the heart rate as the control variable. The aim is to maintain the heart rate of patients within a normal range. To promote the heart function recovery, a baroreflex sensitivity controller has been reported [12]. In this control strategy, the baroreflex sensitivity (BRS) has been used as the control variable. An extreme search algorithm was designed to find the maximum value of the BRS. Then the optimal operating point of the BJUT-II VAD was calculated according to the BRS.

The above-mentioned strategies have been verified in numerical simulation and in vitro, and the results demonstrate that they can regulate the pump according to the change in metabolic demands of their patients. According to Birks’s report [13], the combination of continuous-flow (CF) circulatory support and pharmacological therapy can significantly improve the heart function recovery and the survival rate. Mahmood et al. [14] established a model that combined the cardiovascular system, LAVD and the pharmacodynamics model of sodium nitroprusside. Although studies show the importance of the studies on the interaction between LVAD support and pharmacological therapy, the effect of the pharmacological therapy, such as the angiotensin-converting enzyme inhibitor (ACEI), on the performance of the control strategy of LVAD is neglect by most researchers.

BJUT-II VAD is a novel rVAD, which is developed by the artificial heart research group at Beijing University of Technology [15]. Due to it avoid damaging myocardium and eliminate the percutaneous wires, it is specially fit for long-term support. Therefore, it is important and necessary for BJUT-II VAD to study the interaction between pharmacological therapy and LVAD support under varied control strategies. In the field of pharmacological therapy, captopril which is an angiotensin-converting enzyme inhibitor (ACEI) represents a significant therapeutic advance in the treatment of congestive heart failure [16]. It principally acts by interfering with the local and generalized production of angiotensin II, a hormone that, apart from its vasoconstriction power, also promotes, via aldosterone stimulation, an increased sodium and water retention [17]. Therefore, captopril plays a significant value in clinical treatment of HF.

To achieve this aim, according to the guidelines for the treatment of the acute and chronic heart failure, captopril that is a kind of ACEI is chosen to study the effect of pharmacological therapy on the control strategy of BJUT-II VAD. To mimic the effect of the captopril on the hemodynamic parameters, a pharmacokinetic model of captopril has been established. Then, a combination model of the cardiovascular system model and the pharmacokinetic model of captopril was established. Two control strategies of BJUT-II VAD, which chose the heart rate (HR) [10] and blood assist index (BAI) [18], are chosen as the target control strategies to evaluate the performance of them.

## Methods

### The design of pharmacokinetic model of captopril

**Fig. 1:**
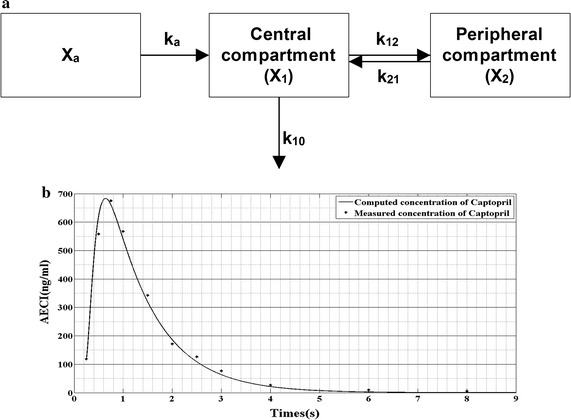
The pharmacokinetic model of captopril. **a** is the scheme of the pharmacokinetic model of captopril; **b** is the waveform of the model. The data of measured concentration of captopril is derived from Ref. [19]

In response to Kelly’s report, the pharmacokinetic model of captopril is consistent with the character of two-compartment model. Hence, the structure of pharmacokinetic model of captopril is shown in Fig. 1a. In this model, it is assumed that the drug delivery is by the first-order pharmacokinetics. According to the theory of pharmacokinetic, the model can be represented by Eqs. (1) to (3).

1 $$\frac{{dX_{1} }}{dt} = k_{\alpha } X_{\alpha } - k_{12} X_{1} + k_{21} X_{2} - k_{10} X_{1}$$

2 $$\frac{{dX_{2} }}{dt} = k_{12} X_{1} - k_{21} X_{2}$$

3 $$\frac{{dX_{\alpha } }}{dt} = - k_{\alpha } X_{\alpha }$$

where *X*, *X*_1_ and *X*_2_ is the dose of captopril tables, the dose of captopril in plasma and the dose of captopril in tissues, respectively. *k*, *k*_12_, *k*_21_ and *k*_10_ represent the coefficients of absorption rate, transport coefficient through plasma to tissues, transport coefficient through tissue to plasma and the coefficient of plasma–drug clearance per unit volume, respectively. Then, the plasma–drug concentration is derived from (1) to (3) as:

4 $$C_{1} (t) = \frac{{k_{\alpha } FX_{0} (k_{21} - k_{\alpha } )}}{{V_{1} (\alpha - k_{\alpha } )(\beta - k_{\alpha } )}}e^{{ - k_{\alpha } t}} + \frac{{k_{\alpha } FX_{0} (k_{21} - \alpha )}}{{V_{1} (k_{\alpha } - \alpha )(\beta - \alpha )}}e^{ - \alpha t} + \frac{{k_{\alpha } FX_{0} (k_{21} - \beta )}}{{V_{1} (\alpha - \beta )(k_{\alpha } - \alpha )}}e^{ - \beta t}$$

where *C*_1_(*t*) is the plasma-drug concentration, *F* represents the absorbed fraction, *V*_1_ represent the apparent volume of distribution.

According to clinical data of plasma-drug concentration [19], shown in Table 1, the parameters in (4) are determined by using data fitting algorithm. Then, the pharmacokinetic model of captopril is derived as:

**Table 1:** The clinical data of the mean plasma-drug concentration of captopril (ng/ml) obtained from 18 patients who oral captopril (50 mg)

Time (h)	Mean value	Standard deviation
0	0.000	
0.25	118.117	141.463
0.5	558.122	326.228
0.75	675.000	398.308
1	576.256	245.560
1.5	342.500	194.182
2	171.782	80.217
2.5	126.100	80.260
3	76.594	50.183
4	26.633	13.936
6	9.397	4.449
8	5.166	2.429

The data is derived from reference [19]

5 $$C_{1} (t) = 170.1996e^{ - 1.08t} + 99419.334e^{ - 9.42t} - 84758.972e^{ - 8.31t}$$

To verify the accuracy of (5), a comparison between the clinical data (measured concentration of captopril) and the data derived from (5) (computed concentration of captopril) is conducted. The result is shown in Fig. 1b. It demonstrates that Eq. (5) can accurately reflect the relationship between time and plasma-drug concentration of captopril.

The sigmoid E_max_ model [20] is chosen as the pharmacodynamic model of captopril, denoted as:

6 $$E(C) = \frac{{E_{\text{max} } C^{s} }}{{C_{E50}^{s} + C^{s} }}$$

where *E*(*C*) is the effect of the captopril in the current concentration, *E*_*max*_ represents the maximum effect, *C*_*E*50_ is the concentration in where the captopril generates their 50% maximum effect, *c* represents the concentration of captopril, *s* is the steepness parameter. In this work, the parameters are set as: *E*_*max*_ = 0.5, *C*_*E*50_ = 300 ng/ml, s = 2.

### The combination model

**Fig. 2:**
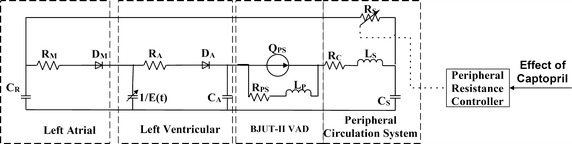
The mathematic model of combination model

In order to evaluate the effect of captopril on control strategy of BJUT-II VAD, a combination model is designed. In this combination model, the pharmacokinetic model of captopril is coupled with a cardiovascular system model. Figure 2 shows the complete electric circuit analogy of the combination model. The model comprises the left atrium, the active left ventricle, BJUT-II VAD, the peripheral circulation system and the controller for the peripheral resistance. The controller adjusts the peripheral resistance according to the output of the pharmacokinetic model and pharmacodynamics model [21], denoted as Eq. (7).

7 $$R_{s} = R_{0} (1 - E(C))$$

where *R*_0_ represents the peripheral resistance without taking captopril; *E*(*C*), which is the output of Eq. (6), is the drug effect of captopril; *R*_*s*_ represents the peripheral resistance at current plasma-drug concentration.

The combination model is described as a nonlinear time-varying lumped parameter model, which is described by a set of differential equations as:

8 $$\dot{X} = A(t)X + B(t)U(X)$$

9 $$A(t) = \left[ {\begin{array}{*{20}c} {\frac{{{ - }\dot{C}(t)}}{C(t)}} & 0 & 0 & 0 & 0 \\ 0 & {\frac{{{ - }1}}{{R_{S} C_{R} }}} & {\frac{1}{{R_{S} C_{R} }}} & 0 & 0 \\ 0 & {\frac{1}{{R_{S} C_{R} }}} & {\frac{{{ - }1}}{{R_{S} C_{R} }}} & 0 & {\frac{1}{{C_{S} }}} \\ 0 & 0 & 0 & 0 & {\frac{{{ - }1}}{{C_{A} }}} \\ 0 & 0 & {\frac{{{ - }1}}{{L_{S} }}} & {\frac{1}{{L_{S} }}} & {\frac{{-}{R_{C} }}{{L_{S} }}} \\ \end{array} } \right]$$

10 $$B(t) = \left[ {\begin{array}{*{20}c} {\frac{1}{C(t)}} & {\frac{{{ - }1}}{C(t)}} & 0 \\ {\frac{1}{{C_{R} }}} & 0 & 0 \\ 0 & 0 & 0 \\ 0 & {\frac{{{ - }1}}{{C_{A} }}} & 0 \\ 0 & 0 & {\frac{1}{{L_{S} }}} \\ \end{array} } \right]$$

11 $$U(X) = \left[ {\begin{array}{*{20}c} {\frac{{\gamma (x_{2} { - }x_{1} )}}{{R_{M} }}} \\ {\frac{{\gamma (x_{1} { - }x_{4} )}}{{R_{A} }}} \\ {P(\omega ,\;Q_{PO}^{{}} )} \\ \end{array} } \right]$$

where γ(*x*) represents the ramp function. The γ(*x*) is used to mimic the unidirectional properties of aortic valve and mitral valve.

12 $$\gamma \left( \varepsilon \right) = \left\{ {\begin{array}{*{20}c} 0 & \quad {\varepsilon \le 0} \\ \varepsilon & \quad {\varepsilon > 0} \\ \end{array} } \right.$$

where *C* is the compliance; *R* is the resistance properties; the left ventricle is represented by the time-varying compliance *C*(*t*) = 1/*E*(*t*). The details of the cardiovascular system model is reported in Refs. [12, 20].

### The control strategies of the BJUT-II VAD

#### The BAI control strategy of the BJUT-II VAD

The energy distribution between the LVAD and native heart is indicated by BAI [11], which is a ratio of power of LVAD and total power of the cardiovascular system, denoted as the Eq. (8).

13 $$BAI(\omega ) = \frac{1}{{T_{C} }}\int_{0}^{{T_{C} }} {\left( {\frac{100U(t)I(t)\eta (\omega )}{{P(t)_{aop} F(t)_{aop} }}} \right)} \;dt$$

where *U*(*t*) is the power supply voltage of the LVAD, *I*(*t*) is the winding current of the pump, *η*(*ω*) represents the efficiency of the pump, which is a function of the rotational speed of the pump *ω*. *P*(*t*)_*aop*_ is the waveform of aortic pressure, and *F*(*t*)_*aop*_ denotes the waveform of the blood flow in the aorta, *T*_*c*_ represents the cardiac cycle; *BAI*(*ω*) represents the blood assistant index, whose unit is %.

**Fig. 3:**
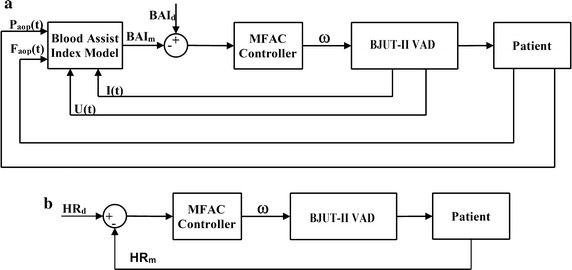
The scheme of control strategies of BJUT-II VAD. **a** The scheme of the BAI control strategy; **b** The scheme of HR control strategy

Figure 3a proposed BAI control strategy. BAI_m_ is assessed and calculated by the pressure (*P*_*aop*_(*t*)), blood flow (*F*_*aop*_(*t*)) of aorta, the power supply voltage of pump (*U*(*t*)) and the winding current of pump (*I*(*t*)) at each heart cycle. Then BAI_m_ and BAI_d_ are used as control input to adjust the rotational speed of the pump. The value of parameters is reported in Ref. [18] and the same method is used in the published paper.

#### The HR control strategy of BJUT-II VAD

According to previous researches of our group, HR indicates the change in metabolic demand. Hence, the HR is chosen as the control variable in the HR control strategy. The aim of this control strategy is to maintain the HR in a normal range. The scheme of the HR control strategy is shown in Fig. 3b. The measured HR (HR_m_) is estimated from ECG signal. The details and the value of the parameters of the control strategy have been reported in Ref. [10].

## Results

In order to evaluate the effect of the captopril on the performance of the control strategy, three numerical simulations are conducted. In the first simulation, the captopril is added into the combination model at 2 s. The accuracy of the combination model without BJUT-II VAD is tested. In the second simulation, the HR control strategy is employed to regulate the BJUT-II VAD. The captopril is added into the combination model at 8 s. The dose of captopril is 50 mg. In the third simulation, the BAI control strategy is used to adjust the pump. The captopril, whose dose is 50 mg, is added into the combination at 10 s.

**Fig. 4:**
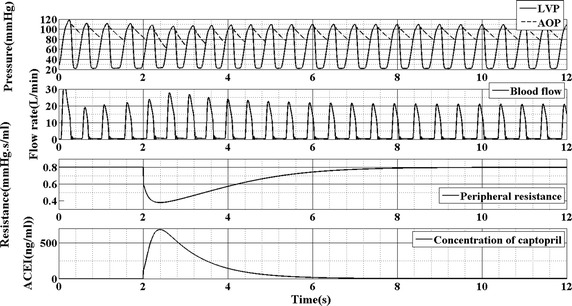
The response of the LVP, AOP, blood flow, peripheral resistance and concentration of captopril without BJUT-II VAD

Figure 4 shows the waveform of left ventricular pressure (LVP), aortic pressure (AOP), blood flow, peripheral resistance and plasma–drug concentration of captopril without VAD support. From this figure, it is seen that aortic pressure and blood flow are consistent with the clinical data. When the plasma–drug concentration of captopril increases, the peripheral resistance decreases accordingly. That means the combination model can mimic the effect of the captopril on the hemodynamics of cardiovascular system.

**Fig. 5:**
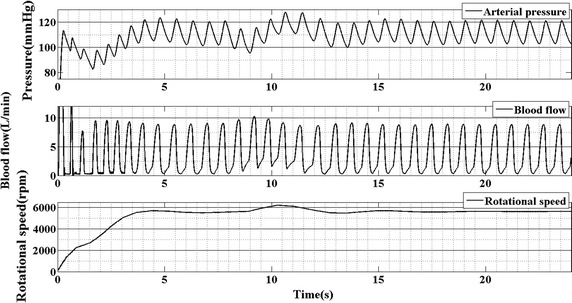
The response of arterial pressure, blood flow and rotational speed supported by HR controller

**Fig. 6:**
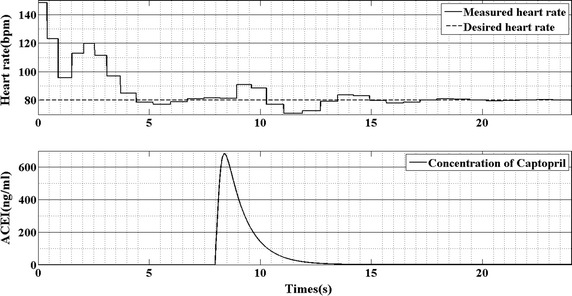
The response of the heart rate and plasma–drug concentration of captopril

To visualize the performance of the HR control strategy, arterial pressure (AP), blood flow and rotational speed are plotted in Fig. 5. The first panel of Fig. 5 is the curve of arterial pressure; the second panel of Fig. 5 is the waveform of blood flow; the last panel of Fig. 5 shows the curve of rotational speed of the pump. It is seen that, when the plasma–drug concentration increases, the arterial pressure decreases accordingly. And the change in arterial pressure leads the heart rate increase (Fig. 6). Then, the rotational speed of the pump increased from 5500 to 6300 rpm to compensate the change in heart rate (Fig. 5 last panel).

**Fig. 7:**
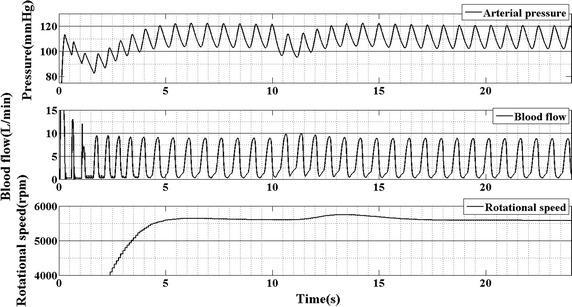
The response of arterial pressure, blood flow and rotational speed supported by BAI controller

**Fig. 8:**
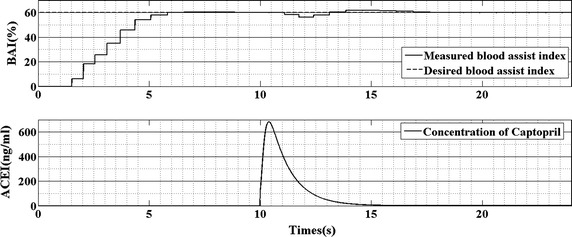
The response of the BAI and plasma–drug concentration of captopril

The response of the BAI control strategy to the captopril is plotted in Figs. 7 and 8. The first panel of Fig. 7 is the curve of arterial pressure; the second panel is the waveform of blood flow and the third panel is the rotational speed of the pump. The change in BAI along with the plasma–drug concentration of captopril is shown in Fig. 8. It is seen that along with the increase of plasma–drug concentration, the mean arterial pressure reduce from 110 to 100 mmHg. This phenomenon leads the BAI decrease from 60 to 55%. Then, the BAI controller increases pump speed to compensate the change in BAI. After 5 s, the BAI track its desired value. During this period, the blood flow also increases because of the increase of rotational speed and the decrease of peripheral resistance.

## Discussion

With the development of LVAD, the “long-term support” has been paid more and more attention by many researchers. In long-term support, the combination of rVAD and pharmacological therapy can significantly improve the heart function recovery and the survival rate of heart failure patients [22]. For instance, Birks et al. [13] report their recent experience using the combination of continuous-flow (CF) circulatory support and pharmacological therapy to treat advanced heart failure in patients requiring LVAD support. The results demonstrate that there is about 60% study cohort met criteria for LVAD explantation and about 50% who survived the perioperative period demonstrated sustained recovery over 56–1112 days of follow-up. In 2006, Harefield recovery study demonstrates that data regarding combined pharmacological and mechanical support were available [23]. The authors reported that 75% of patients receiving clenbuterol could undergo LVAD explantation. Therefore, the combination of the pharmacological therapy and LVAD is significantly important for long-term support. And the study of the effect of pharmacological therapy on the performance of control strategy of LVAD is the basis of design combination therapy.

The critical of evaluating the control strategy of LVAD is to verify its response to the changing in hemodynamic parameters. However, traditional way is to set the variation of hemodynamic parameters by operator’s idea. For instance, in Ref. 13, the peripheral resistance is changed by step function. That is the peripheral resistance in this work occurred jump. In Karantonis’ report, the peripheral resistance is set to change in accordance with linear law [24]. However, in clinical, the hemodynamic parameters are usually changed by drugs. For instance, the captopril has been widely used to reduce the peripheral resistance. Therefore, on one hand the pharmacokinetic model is introduced into the evaluation of control strategy of rVAD can provide a more realistic hemodynamic status for the evaluation of control strategy. On the other hand, it can give doctors more intuitive guidance on how to manage the pharmacological therapy under rVAD support.

Figure 4 shows the waveform of left ventricular pressure (LVP), aortic pressure (AOP), blood flow and the peripheral resistance. It is seen that, along with the increase of plasma-drug concentration of captopril, the peripheral resistance is reduced accordingly. Because of the decrease of peripheral resistance, the blood flow increase accordingly (Fig. 4 second panel). That means the captopril can reduce the afterload of the left ventricle, which is consistent with the clinical data. Hence, combination of rVAD and captopril to treat advanced heart failure is significant. Moreover, the results of simulation demonstrate that the combination of pharmacokinetic model and the lumped parameter model of cardiovascular system can mimic the effect of drug on the hemodynamics of cardiovascular system. This is a novel method for researchers to evaluate the performance of control strategy.

From Fig. 6, it is seen that when the concentration of captopril increase, the heart rate will increase accordingly. That because the captopril reduces the afterload of the left ventricle, which will reduce the arterial pressure (Fig. 5 first panel). According to Chang et al. [10], the heart rate in the cardiovascular-baroreflex system is regulated by the baroreflex system. When the arterial pressure decreases, the frequency of spikes in the afferent fibers will reduce. The sympathetic nerves and vagal fibers choose the frequency of the spikes in the afferent fibers as the input. When the frequency reduces, the sympathetic will be activity and on the contrary, the vagal will be inhibition. This phenomenon results in the increase of the heart rate.

Figure 8 plots the waveform of BAI and plasma–drug concentration of captopril. It is seen that along with the increase in plasma–drug concentration of captopril, the BAI will reduce. That because captopril will reduce the peripheral resistance. This will reduce the differential pressure of the BJUT-II VAD and change the energy distribution between the native heart and BJUT-II VAD.

The above-mentioned simulation results demonstrate that the captopril can affect the hemodynamics of cardiovascular system by changing the peripheral resistance. Due to the control strategy of rVAD chooses the hemodynamic parameters as the control variables to evaluate the metabolic state of patients; the captopril will directly affect the performance of the control strategy. Hence, it is important for researchers to evaluate the performance of control strategy under pharmacology therapy. The combination model established in this work provides a novel method to achieve this aim. Moreover, both of the control strategies evaluated in this work can respond to the change in hemodynamic parameters and maintain the cardiovascular system in a normal range. That means the control strategies evaluated here can be applied for patients who under pharmacological therapy.

In this work, the pharmacokinetic model is established based on the single dose pharmacokinetic model therapy. According to the guidelines for the treatment of the acute and chronic heart failure, the captopril will be taken repeatedly. Hence, the multi-dose pharmacokinetic model should be established. Also, Viecili [25] reported that captopril alone produced an increase in cardiac index and a decrease in systemic vascular resistance and pulmonary capillary wedge pressure. In the future, pressure of pulmonary can be considered for captopril.

According to the pharmacokinetic theory, the individual differences of pharmacokinetic parameters are very obvious. Hence the researchers should determine the pharmacokinetic parameters of patients’ own, and established the personalized combination model for patient. Then the personalized combination therapy strategy will be designed based on the combination model. This work will be conducted in the future.

## Conclusion

The pharmacokinetic model of captopril, based on clinical data, is established. The combination model that includes a cardiovascular system model and the pharmacokinetic model is proposed. The simulation results demonstrate that the combination model can reflect the effect of captopril on the hemodynamics of cardiovascular system. The BAI control strategy and HR control strategy of BJUT-II VAD are employed to evaluate the effect of pharmacological therapy on performance of control strategy. The results showed that captopril could affect the performance of BAI control strategy and HR control strategy. Both of control strategies can respond to the change in hemodynamic parameters caused by captopril. The combination model proposed here provides a novel method for researchers to evaluate the performance of rVAD controller.
